# Trends in adult physical fitness in China: an analysis of national monitoring reports (2000–2020)

**DOI:** 10.3389/fspor.2025.1578817

**Published:** 2025-05-15

**Authors:** Huabin Hang

**Affiliations:** School of Physical Education, Nanjing University of Technology, Nanjing, China

**Keywords:** physical fitness, national monitoring, China, physical activity, adults

## Abstract

**Background/Objectives:**

To summarize the results of China's five national monitoring reports (CFNMR) on physical fitness (PF) for adults. The Government of China has taken a systematic administrative approach to a creative environment building, and has conducted five national physical fitness monitoring, with a sixth already started in 2024.

**Methods:**

CFNMR included indices, rates, test indicators, and questionnaire indicators collected between 2000 and September 2024. Data were collected (every 5 years), analyzed (every 3 years) and published (government announcements and reports) by the Monitoring Center of the General Administration of Sport of China. Adults’ data were divided into Group A (20–39 years) and Group B (40–59 years). In this study, once the database was established using government reports as the standard, the indicators were categorized, counted, and descriptively analyzed using EXCEL.

**Results:**

Group A: Indices fluctuated up 2.98 (2010 highest 102.98), and rates fluctuated up 2.40% (2005 highest 89.3%). Group B: Indices continued to decline 0.23 (2014 lowest 99.77), and rates fluctuated up 3.00% (2019 highest 90.6%). Test and questionnaire indicators show different structural characteristics, but obesity (7.33%) and overweight rates (5.88%) continue to increase.

**Conclusion:**

Adult physical fitness is improving, but physical activity is decreasing. The results of the overall growth shown by the tested indicators do not, however, represent the formation of well-functioning mechanisms. Obesity is an ongoing and growing problem that requires constant attention. It should consider adding a physical literacy monitoring component, utilizing public goods attributes, to promote sustainable change and reach more adults.

## 1 Introduction

Physical inactivity is a growing problem worldwide (1), including China (2), where the government has invested in monitoring and intervention programs since 2000. The aim is to collect comprehensive data that will enable the Government to determine the direction of administrative and policy efforts and the development of interventions, and to disseminate the results of the tests nationwide (3). Once the results of the monitoring are available to General Administration of Sport of China (GASC), two reports (Report and Bulletin) are produced (4). The Physical Fitness Bulletin becomes a powerful advocacy tool (5). The National Surveillance Report becomes a data resource for the development of interventions in search of interventions, and scientific research and policy form a mutually supportive environment (5). All become a strategy to address the crisis of physical inactivity in adults to enhance their physical fitness (PF).

In China, levels of physical inactivity among adults are concerningly high (6). Since the first 2000, about one in five adults has been physically inactive, making it one of the least active countries in the world (1, 6). Therefore, there is a need to develop comprehensive monitoring and awareness-raising tools and to follow the times with targeted monitoring mechanisms, such as nationally monitored PF bulletins (7). China's five national monitoring reports (CFNMR) with adult data divided into Group A (20–39 years) and Group B (40–59 years) released since 2000 show that the national physical fitness attainment rates have been increasing 3.3% (87.1% in 2000, 87.2% in 2005, 88.9% in 2010, 89.6% in 2014, and 90.4% in 2020) (8–11). However, the results for the age subgroups show a decreasing trend in rates with age and, Adult B rates are the most relatively low (88.5% ranked 1 in 2000, 87.8% ranked 3 in 2005, 87.6% ranked 3 in 2010 88.1% ranked 3 in 2015, 90.6% ranked 3 in 2020) (12–14). Over the past two decades, China has continuously adjusted its policies and increased its infrastructure investments (15), and a national monitoring system has been developed (16), but improvements in adult fitness need to be further observed across the cross-section. Physical literacy, was a new concept introduced by Whitehead in 1993 (17–20). Physical literacy as a philosophy is an effective strategy, for addressing the global phenomenon of sedentary behavior and physical inactivity (21–25). Physical literacy serves as a proximal measure of an individual's physical activity (26), which is assessed, instructed, and intervened (27), to improve individual fitness (28, 29), establish a health ecology (30), and mitigate chronic disease and obesity rates (31–34), forming a PL public health system (35). The role of physical literacy in promoting PF in adults has received attention from research scholars (36–38). Physical literacy becomes an indicator in the Healthy China 2030 program (39).

CFNMR has been well-received as an act of positive health policy (14). The Chinese government has engaged in systematic creative environment building, for example, Several Opinions of the State Council on Promoting the Development of Health Service Industry in 2013 (3). In addition, a national monitoring system has been formed, including an indicator subsystem, a network subsystem and monitoring centers at the provincial, municipal and county levels (16). Considering that the monitoring reports have the potential to guide future national processes to promote adult PF, separate questionnaires were administered in each monitoring process (2000 Regional, 2005 Ethnic, 2010, 2014 and 2020 Occupational) (8–11). The World Health Organization (WHO) Global Action Plan for the Prevention and Control of Non-communicable Diseases, released in 2013, set 2025 as a key assessment point, requiring countries to achieve a “zero obesity growth rate” by 2025, and to develop standardized guidelines for weight management (40–44). Chinese researchers and scholars have conducted cross-sectional and cohort studies in adult populations, exploring topics such as BMI with disease and geographic differences (45–48).

The purpose of this study was to summarize the findings of CFNMR on PF as a valid complement to national monitoring in China, and the process descriptive statistics of the changes between the five data sets were the initial calculations for the data collation. The completeness of the database is a factor in the impact of surveillance on health as a regional policy.

## 2 Methods

### 2.1 Materials and samples

CFNMR included indices, rates, test indicators (3 primary, 20 secondary, and 7 derived indicators), and questionnaire indicators (demographic characteristics, living habits, physical activity, and sports) (2). More details of CFNMR methodology can be found in (Tables 1, 2). The monitoring indicators are a complete set of indicators screened and determined by experts in 1996–1999 by combining the research results of national PF of different groups over the years at home and abroad, and by applying the principles and methods of mathematical statistics, through literature research, expert argumentation and experimental research (3). The National Center for National Physical Fitness Monitoring (NCNPFM) collected and analyzed these data, and based on the definitions and revised benchmarks of the National Physical Fitness Test Standards (NPFTS), the test scoring criteria for the adult group, with a 5-point scoring system for individual measurements, the same scoring criteria for the same age group, and four levels (excellent, good, passing, and failing) (8–11). During the Third National Physical Fitness Monitoring in 2010, “quality control” appeared for the first time, and quality control of data was carried out (10). It is about mainly through a team of supervisors, retesting of abnormal data, retesting of samples, data entry (error rate 0.5‰ or less), and data acceptance (deletion of data appearing to be out of the range of the logic program) (10). GASC is the national administrative organ of China. The verification of data is guaranteed through the NCNPFM by GASC (13). Indicators for which data are not sufficiently directly available are rated as incomplete (INC). The validity of data is guaranteed through the Sports Science Institute by NCNPFM (4). The inevitability of data is guaranteed through the National PF Research Center by Sports Science Institute (4, 13).

**Table 1:** Test indicators and overall evaluation of Chinese adults (2000–2020).

Indicators	Items	Group A	Group B	Overall evaluation
Physical indicators^a^				★★★★★●
	Height	○	○	
	Sitting height	○	○	
	Body weight	○	○	
	Bust	○	○	
	Waist	○	○	
	Hips	○	○	
	Upper arm skinfold thickness	○	○	
	Abdominal skinfold thickness	○	○	
	Scapular skinfold thickness	○	○	
Functional indicators^a^				★★★★★
	Quiet tulse (heart rate)	○	○	
	Blood pressure	○	○	
	Spirometry	○	○	
	Step test	○	○	
Fitness indicators^b^				★★☆☆☆
	1 min sit-ups	Female		
	Grip strength	○	○	
	Strength	○		
	Long jump	○		
	Push-ups	Male		
	Stand on one leg with eyes closed	○	○	
	Selection of reaction time	○	○	

○, both males and females; ★★★★★, the base period value in 2000; ●, growth; ☆, decline. Group A, 20–39 years old, Group B, 40–59 years old.

^a^ Assessment of changes in national physical fitness monitoring and rating.

^b^ Star rating based on the 2022 bulletin.

**Table 2:** Calculated statistics of variance for group adults (2000–2020).

Groups		2000	2005	2010	2015	2020
(A) Statistical table of five monitored NPFAR (unit: %)						
Adults						
	Group A	86.90	89.30	88.40	89.00	87.20
	Group B	88.50	87.80	87.60	88.10	90.60
Groups all		87.10	87.20	88.90	89.60	90.40
(B) Statistical table of the samples for the five monitoring campaigns (unit: persons)						
Adults						
	Group A	1,51,656	1,63,448	1,55,054	1,46,703	1,21,928
	Group B	76,909	82,272	77,487	76,081	60,964
Groups all		74,747	81,176	77,567	70,622	60,964
(C) Statistical table of differences in groups adults (within and outside groups) at five monitoring sessions						
UA-B		9.48**	9.52**	4.85**	5.41**	18.90**
	Precedence	Group B	Group A	Group A	Group A	Group B
UA-ALL		0.00	0.00	0.00	0.00	0.00
	Precedence	Group all	Group all	Group all	Group all	Group all
UB-ALL		0.00	0.00	0.00	0.00	0.00
	Precedence	Group B	Group B	Group all	Group all	Group B

Very significant difference**, *U* > 2.58, *P* < 0.01 (Very significant difference); *U* < 1.96, *P* > 0.05 (No significant difference).

In addition, in 2000, the Government drew on international experience to define the concept of physical fitness, and form a system of indicators to facilitate international comparisons (11). Finally, the grades were also converted into a Composite National Physical Fitness Index (CNPI) and the National Physical Fitness Attainment Rate (NPFAR). CNPI is 100.00 for the first time in 2000, to facilitate more visual comparison, dissemination and interpretation in the country. The adult group was divided into two groups for the calculation, Group A (20–39 years old) and Group B (40–59 years old) (10). GASC, in conjunction with 10 other departments, monitors the situation every 5 years, with each monitoring lasting 3 years, issues monitoring reports and bulletins, and reaches a consensus on measures and actions to improve the future grade of each indicator [National Physical Fitness Test indicators (NPFTI) and National Physical Fitness Questionnaire indicators (NPFQI)] (13).

In accordance with the criteria for ethical review, our study was a descriptive secondary analysis based on publicly available data, and therefore did not require ethical approval.

### 2.2 Research design

Since January 2024, three databases have been constructed: government reports, government bulletins and scientific articles. Government reports, the inclusion criteria are based on publicly published thematic reports and academic books. Government announcements, the criteria for inclusion are the public posting of topical blogs on government function websites, such as GASC. Scientific articles, searched on the China Knowledge Network (Chinese database) and five English databases (ProQuest, ERIC, Science Direct, Scopus, and Sport Discus), were included. The criteria for the inclusion of scientific articles were (a) the title contained the dual keywords of Chinese national physical fitness monitoring and adults; (b) the period was from 2000 to 2024; and (c) the full text could be read and downloaded. Here, the researcher needs to show that scientific articles are effective supplements to government reports and announcements. However, scientific articles that are not based on data published in government reports, will not be included in this study. Once the database was established, the indicators were categorized and counted using EXCEL, using government reports as the standard. Separate screens for SHEET A and SHEET B were created based on the adult AB group, and the indicators were filled in separately. The statistical data of this study contains CNPI and NPFAR. NPFTI (Table 1) and NPFQI, based on materials for descriptive combing.

If unpublished data are encountered, they are marked as Null Frequency Complete (NFC). If the data are shown to be relatively continuous by year, they are included in the system analyzed, otherwise specific reasons are given.

Our study was a descriptive secondary analysis. The analytical steps of the study were set up to perform initial descriptive statistics, followed by analysis of variance (if the data were continuous).

### 2.3 Data calculation

Our study is a secondary calculation for the five national physical fitness monitoring data. First, the study used EXCEL for data collection and organization to determine the continuity of the data. In the second step, using an EXCEL table, the figures were created to facilitate a more visual representation of the findings. In the third step, the variability calculation is performed for continuous data using an EXCEL sheet. Setting the criteria for calculation, the statistic U was calculated according to the formula [ABS ()/SQRT ()], and the conclusion of the statistical test was made according to the *U* value on the normal distribution table (*P* = 0.01, *U* = 2.58; *P* = 0.05, *U* = 1.96) (2).

## 3 Results

In accordance with the study design, we carried out descriptive secondary analysis of the adult group, considering both serial and control observations. We observed: the fluctuation of CNPI (2.28 increases in group A and 0.23 decreases in group B); the fluctuation of NPFAR (group A folded up, group B declined first and then grew) and the difference [significant difference between group AB (*P* < 0.01), no significant difference outside the group (*P* > 0.05)]; the trend of NPFTI (physical indicators continued to grow, functional indicators were basically maintained, and fitness indicators continued to decline).

We need to recognize that firstly from the information we collected, we found that not all of the information is continuous, and secondly our study analyzed the NPFAR (linkage information) for correlation and difference.

Through the compilation of data, our study found that NPFQI does not have a specific data, but only relevant descriptive statistics in government bulletins, which may be an absence. Some researchers have argued that the variability of results in socio-demographic variables (gender, age, and type of work, etc.) is still present (2). In addition, the results of our research statistics found that NPFTI has partial descriptive statistics in government bulletins, so we made a summary of the salient issues (labeled as INC in this study). CNPI, some data are missing, but relative continuity statistics are available. NPFAR, all data are continuous and complete.

### 3.1 CNPI

**Figure 1:**
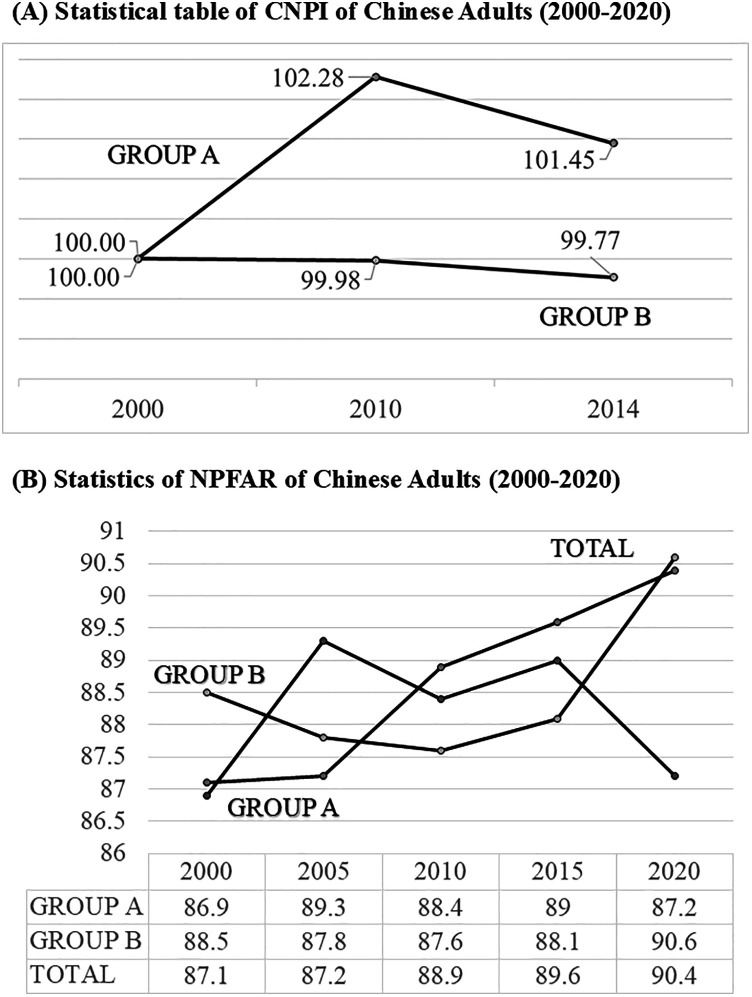
Composite national physical fitness index (CNPI) and the national physical fitness attainment rate (NPFAR) statistics of Chinese adults (2000–2020). **(A)** Statistical table of CNPI of Chinese adults (2000–2020). **(B)** Statistical of NPFAR of Chinese adults (2000–2020).

Group A: fluctuated up 2.98 (100 in 2000, NFC 2005, 102.28 in 2010, 101.45 in 2014, NFC 2020), Group B: continued to decline 0.23 (100 in 2000, NFC 2005, 99.98 in 2010, 99.77 in 2014, NFC 2020). Figure 1A Considering, that there is more missing data, no difference in difference calculation was done in this study.

CNPI is a comprehensive indicator of physical health and is used to assess the overall health of a population (2). The change in CNPI is relevant in the overall level of economic development of the country (39). China has been reforming and opening since the 1990's (33). Economic development has raised people's standard of living, bringing with it richer food that provides more nutritional value, but also sedentary behavior and physical inactivity (39–41). So it could be that the increase in nutrition is causing the CNPI to be on the rise in the adult A group; and the multiple factors of excess nutrition and sedentary behavior and physical inactivity are bringing about a continued decline in the CNPI in the adult B group. This trend is a signal to public health that both the increase in adult Group A and the decrease in Group B require further attention.

### 3.2 NPFAR

Group A: fluctuated up 2.40% (87% in 2000, 89% in 2005, 88% in 2010, 89% in 2014, 87% in 2020). Group B: fluctuated up 3.00% (89% in 2000, 88% in 2005, 88% in 2010, 88% in 2014, 91% in 2020). Grade statistics are all NFC (Figure 1B).

*U*-test [ABS ()/SQRT (), (2)] was applied to the data in an EXCEL table, to verify the differences in NPFAR (in-group and out-of-group) of the adult groups on five occasions of monitoring. For Groups AB, the results of all the five occasions were significantly different [*U* > 2.58, *P* < 0.01, (2)], with a minimum of 4.85, and three times preceded in Group A, and two times preceded in Group B. The results were calculated for Groups A and B, respectively, and for Groups ALL. Group A and Group B were calculated separately with Groups ALL, and both were non-significant [*U* < 1.96, *P* > 0.05, (2)], and Group A preceded all groups only once, and Group B preceded Groups ALL on three occasions (Table 2).

NPFAR is a distribution phenomenon of the physical condition of the population within the group (2). The within-group differences illustrate that there is a difference between the adult AB groups. The fact that adult group A took precedence more often than group B is more likely to be an age advantage. The phenomenon of no difference between groups indicates that the adult AB group is homogeneous with all groups. Group B took the lead 3 times and Group A took the lead 1 time, indicating that Group AB had a surplus of PF passes over all groups.

### 3.3 NPFTI

Physical Indicators: Continuous growth. Adults' weight, waist circumference, hip circumference and body fat percentage increase and then decrease with age, but the overall trend is increasing. Overweight and obesity rates have increased significantly in the Chinese adult population, reaching 35% and 14.6% in 2020. Functional Indicators: Basically maintained. The mean values of the indicators for adults show a decreasing trend with age. There is a small increase in blood pressure, and lung capacity is basically maintained. Fitness Indicators: Showing a continuous downward trend. Maximum muscle strength, represented by grip strength and back strength, showed an increase and then a decrease with age, while all other physical fitness indicators showed a decreasing trend with age (Table 1).

NPFTI is a materialization of the body's constitution (2). The growth of physical indicators, the decline of functional indicators, and the decline of fitness indicators are strongly age-specific (38, 39). Since we did not have access to more detailed data, we were unable to calculate the magnitude of the decline and the rate value (in difference comparison). However, from the results obtained, the continued increase in overweight and obesity is like the results of previous research scholars (45–48). Obesity rates in the adult group are alarmingly high and require more systematic and comprehensive policy attention and guidance.

## 4 Discussion

This manuscript summarizes the findings of CFNMR on adult PF.

The overall performance of adult PF, both in terms of CNPI and NPFAR, suggests little change (49–52). All Group A grades were slightly higher than Group B grades, but this was not reflected in improvements in overall PF and related behaviours, and was more likely due to age factors. The results of the study show that overweight and obesity rates continue to increase in the Chinese adult population (Table 1), despite government efforts to promote physical activity and healthy eating (Table 3). These findings are consistent with the theory of nutritional transition (40), which suggests that developing countries are undergoing a change in dietary and physical activity patterns (29), leading to an increased risk of non-communicable chronic diseases.

**Table 3:** Policies related to adult fitness issued by various government departments.

Years	Government departments	Focus
1993^A^	Administration of Sport of China^a^	Formulation of Physical Fitness Measurement Standards for Chinese Adults
1996^A^	Administration of Sport of China^a^	Implementation of Physical Fitness Measurement Standards for Chinese Adults
1997^A^	Administration of Sport of China^a^	Conducting adult physical fitness monitoring
2000^A^	State General Administration of Sport, Ministry of Education, Ministry of Health and et al. 10 departments^b^	First national physical fitness monitoring; development of National Physical Fitness Measurement Standards for young children, adults and elderly
2005^A^	State General Administration of Sport, Ministry of Education, Ministry of Health and et al. 10 departments^b^	Second National Physical Fitness Monitoring
2008^B^	Ministry of Health^b^	Health Literacy for Chinese Citizens—Basic Knowledge and Skills (2008)
2010^A^	State General Administration of Sport, Ministry of Education, Ministry of Health and et al. 10 departments^b^	Third National Physical Fitness Monitoring
2010^B^	Ministry of Education^b^	The National Program for Medium- and Long-Term Educational Reform and Development (2010–2020) was issued, proposing that the promotion of the healthy growth of students should be the starting and ending point of all school work
2012^B^	Ministry of Education, National Development and Reform Commission, Ministry of Finance, General Administration of Sport of China^b^	Issuance of Several Opinions on Further Strengthening School Sports Work; the physical and mental health of young people and their physical fitness is a sign of the progress of civilization in society; strengthening school sports, enhancing the physical fitness of students, and building a strong human resources country
2013^A^	State Council^a^	Issuing the State Council's Opinions on Promoting the Development of the Health Services Industry; coordinating the promotion of reforms in related areas, such as the equalization of basic public health services; and widely mobilizing social forces and taking multiple measures to develop the health services industry
2014^A^	State General Administration of Sport, Ministry of Education, Ministry of Health and et al. 10 departments^b^	Fourth National Physical Fitness Monitoring
2014^A^	State Council^a^	The State Council's Opinions on Accelerating the Development of the Sports Industry and Promoting Sports Consumption, which elevated national fitness to a national strategy, explicitly proposed “improving the national fitness monitoring system”, “providing people with fitness testing services”, and “regularly publishing national fitness monitoring reports”. It also explicitly proposes “improving the national fitness monitoring system”, “providing people with fitness testing services”, and “regularly releasing reports on national fitness monitoring”
2015^B^	Ministry of Health^a^	Health Literacy for Chinese Citizens—Basic Knowledge and Skills (2015)
2016^B^	State Council^a^	The Opinions on Strengthening School Sports to Promote the All-round Development of Students’ Physical and Mental Health were issued, for the first time explicitly mentioning the comprehensive improvement of students’ sports literacy at the national level
2016^B^	State Council^a^	The biggest highlight of the issuance of the National Fitness Plan (2016–2020) lies in the “breakthrough understanding” of national fitness, making it a powerful support for the construction of a healthy China and a national business card for building a moderately affluent society in all aspects
2016^A^	State Council^a^	The Outline of the “Healthy China 2030” Plan explicitly calls for “carrying out national physical fitness tests, improving the physical fitness and health testing system, and developing and applying national physical fitness and health monitoring data”, and puts forward specific requirements for the pass rate of the National Physical Fitness Measurement Standard and the excellence rate of the National Student Physical Fitness Standard; and puts forward requirements for “improving physical literacy”. The Outline explicitly calls for “carrying out national physical fitness tests, perfecting the physical fitness test system, and developing and applying national physical fitness monitoring big data”, and puts forward specific requirements for the pass rate of the National Physical Fitness Measurement Standards and the excellence rate of the National Physical Fitness Standards for Students, as well as the requirement of “improving physical fitness.”
2018^B^	State Council^a^	Opinions on promoting the development of “Internet + Medical Health”; setting up an Internet information platform; carrying out remote health consultation and health management services
2019^A^	State General Administration of Sport, Ministry of Education, Ministry of Health and et al. 10 departments^b^	Fifth National Physical Fitness Monitoring
2019^A^	State Council^a^	The Outline for the Construction of a Strong Sporting Nation was released; it states that by 2020, the physical literacy and health level of the entire nation will continue to improve; by 2035, the physical literacy of young people will be significantly improved; and by 2050, the physical literacy of the people will be among the highest in the world.
2020^B^	State General Administration of Sport and Ministry of Education^b^	The Opinions on Deepening the Integration of Physical Education and Sports and Promoting the Healthy Development of Young People were issued, prioritizing the promotion of the all-round development of young people
2020^B^	State Council^a^	The issuance of the Opinions on Comprehensively Strengthening and Improving School Physical Education in the New Era; strengthening school physical education is a key means of addressing the declining quality of health
2020^A^	Shanghai, Suzhou, Zhejiang and Anhui Sports Bureau^b^	Joint issuance of Several Opinions on the High-quality Development of Sports Integration in the Yangtze River Delta Region; exploration of the institutional system and path model for the integrated development of regional sports
2022^A^	State Council^a^	Issuance of a circular on the 14th 5-Year Plan for National Health; putting people's health in the first place, providing all-round, full-cycle health services for the public, and continuously improving people's health standards
2024^B^	Ministry of Health^a^	Health Literacy for Chinese Citizens—Basic Knowledge and Skills (2024)
2024^A^	State General Administration of Sport, Ministry of Education, Ministry of Health and et al. 10 departments^b^	Sixth National Physical Fitness Monitoring

^A^ Directly related, ^B^Indirectly related, ^a^Independently managed, ^b^Jointly managed.

China has worked very hard to increase physical activity opportunities for adults to boost their PF. China intervenes administratively in the monitoring, yet systematic, coordinated and coherent mechanisms for sustainable change must be accomplished through 10 different departments. It is very good that after 20 years of development, China has a relatively independent system for monitoring PF. Further efforts should be made to appropriately integrate policies from the sports, education, social development, and public health sectors. Using the public goods attribute of monitoring, systematized measures through physical literacy may be a good political strategy. As of December 2024, the government issued specific recommendations related to improving PF, which are summarized in Table 3.

### 4.1 Public goods strategy

The Chinese government has promoted the monitoring of PF in adults in an administrative manner, as can be seen from the first government subject document (In July 1996, Decree No. 22 of the national Physical and Sports Commission issued and implemented the Measures for Implementing PF Measurement Standards for Adults in China) (Table 3). In addition, this monitoring is a systematized presence, while there seems to be no consideration of integrating the qualities between them (49–52). Based on the current CFNMR, we find that it seems possible to redefine government-provided adult PF monitoring services in terms of public good attributes. Given the public health limitations of adult PF monitoring services, public goods policies may be more conducive to improving adult PF and promoting physical activity (2).

Combined with the results of the data obtained in our study, it suggest that the adult group's PF needs to be improved. It is the changes in CNPI (continued decline), the variability in NPFAR (between and within groups), and the changes in NPFTI (especially the continued increase in overweight/obesity rates) in the adult B group. China has been promoting public health policies in a systematic manner through administrative forces. As can be seen from Table 3, there are 15 administrative policies directly related to national physical fitness monitoring (PF/health improvement), 10 indirectly related to it, and 16 independently managed and 9 joint managed. China's national physical fitness monitoring has been characterized by the two main features of non-otherness and non-competitiveness of public goods (53). The formation of public products for national physical fitness monitoring can lift the restriction of every 5 years as a testing cycle. It also can enhance the frequency of service provision, which is conducive to the public's PF enhancement, and can even set up a dynamic database for time-to-time monitoring.

### 4.2 Physical literacy conceptual strategies

The implementation of NPFTS promulgated by GASC has facilitated the monitoring of current adult PF. This monitoring helps to obtain nationally representative data on the numbers of PF indicators. Government functionaries will release reports and bulletins after each monitoring. However, no specific data on the questionnaire indicators were published. We were not able to calculate the structural factors of PF that are related to body mass. This could be one of the endeavours of future work in public health policy. In addition, this study recommends that policies related to measuring physical literacy be developed as soon as possible, because the Chinese government's Vision 2030 program will already incorporate the indicator (54), and because of its importance to PF promotion throughout the lifespan.

National Physical Fitness Monitoring data for the adult group indicate that PF needs further improvement and enhancement. From the perspective of the individual audience, PF is the material foundation of health and a prerequisite for the body to maintain good health. Physical fitness monitoring is a way to understand the phenomenon of one's own PF, and only by understanding the weaknesses of one's PF can one achieve a logical approach to improve and enhance PF. Physical literacy consists of four attributes, emotion, cognition, skills and behavior (55), and provides a mindset for individual PF/health improvement and enhancement. Based on national physical fitness monitoring and guided by the physical literacy indicators of Healthy China 2030, individuals can improve sedentary behaviors and physical inactivity through the enhancement of physical literacy concepts with the four attributes of emotion, cognition, skills and behaviors, which can effectively enhance PF and curb the phenomenon of overweight/obesity.

### 4.3 Implementation theories

Using the implementation theories of scholars like Pressman and Wildavsky (56), Van Meter and Van Horn (57), Bardach (58), and others (59–63) offer a well-established framework to understand why gaps exist between policy goals and real-world outcomes. The fitness monitoring system in China reveals notable deficiencies, as underscored by traditional implementation theories (56–63). The parallel existence of high CNPI scores alongside increasing obesity rates is elucidated by Pressman and Wildavsky's complexity theory (56), which further underscores implementation challenges across all government tiers. Van Meter and Van Horn's framework (57) reveals incomplete objectives, as physical literacy remains absent despite improved metrics. Bardach's implementation games highlight problematic siloing between health, education, and sports departments (58). Berman and Grindle emphasize how standardized national tests may overlook regional variations and sociocultural contexts (59, 60). Lipsky's street-level bureaucracy framework underscores how crucial frontline workers are in fostering lasting engagement in physical activity (61). Nakamura and Smallwood's top-down and bottom-up perspectives help us understand the difference between changes in individual behaviors and broader national evaluations (62). Ultimately, the policy design framework presented by Mazmanian and Sabatier elucidates why China's strong measurement systems (CNPI and NPFAR) have not yielded improved health outcomes (63). It indicates that for fitness policies to succeed, they need to not only gather extensive data but also implement it across various sectors, tailor it to local contexts, and employ strategies that connect assessment to enduring behavioral change (56–63).

The results of CFNMR, based on implementation theory, suggest that the gap between policy implementation (national health goals) and outcomes (undifferentiated change) is an existence that deeply suggests that society/policy (systematic presence of CFNMR), the economy (regional disparities/jointness of sectors), and the culture (indicators of physical literacy/trends in obesity) are still in need of further improvement and upgrading.

### 4.4 Contributions and limitations

Contributions: Our study provides descriptive secondary analysis for five physiological cross-sectional observations of physical fitness monitoring results in the adult group. This study can form a data basis for policy issues for improvement, and it can also lay the foundation of the database for further, more detailed studies. This study contributes to the literature by providing a comprehensive synthesis of the results of the national physical fitness monitoring reports in China, identifying important trends and highlighting the need for more effective interventions to combat physical inactivity and obesity.

Limitations: This study is limited by its descriptive nature and the use of secondary data, which prevents the establishment of causal relationships and the control of data quality. Furthermore, the current CFNMR includes representative data as well as results for most of the indicators, but remains incomplete (reports lacks relevant data for the questionnaire indicators). In addition, comparisons between different versions of the Chinese Adult PF should be interpreted with caution, as some of the indicator values were better represented only after differential calculations.

### 4.5 Conclusion and future directions

The results of the study show that, although the CNPI has shown a slight improvement between 2000 and 2020, overweight and obesity rates have increased significantly in the Chinese adult population, reaching 35% and 14.6% in 2020. These findings suggest that existing health promotion policies may not be sufficient to combat the problem of obesity.

Most of the indicators from CFNMR have not changed much overall, but overweight and obesity are expanding. These findings suggest that public good attributes should be considered to improve public services and to improve the perception of individual PF.

China has a systematic monitoring system, but there is still a gap between PF/physical literacy and public health policy. It is recommended that the physical literacy indicators of Healthy China 2030 be used as a guideline to promote the concept of physical literacy in a comprehensive and systematic way.

In the face of the global crisis of physical activity, China, in the form of government management, is committed to improving the quality and impact of public health policies, and has been striving to implement a comprehensive system. Our study is suggesting that national physical fitness monitoring should be used as a system of public health products and to enhance the provision of public services.

## Data Availability

The original contributions presented in the study are included in the article/Supplementary Material, further inquiries can be directed to the corresponding author.
